# Publisher Correction to: BMC Plant Biology, Volume 20, supplement 1

**DOI:** 10.1186/s12870-020-02802-9

**Published:** 2021-01-15

**Authors:** BMC Plant Biology

**Affiliations:** London, UK

Supplement 1 published in Volume 20 of BMC Plant Biology contained several incorrect 'received dates'^1^. These errors were introduced during the production and publication process. In this correction article the incorrect and correct dates are published. The original articles [1–10] have been updated.

**Table:** 

Publication	Original (incorrect) received date	Current (correct) received date
Genome-wide association study of leaf rust resistance in Russian spring wheat varieties	29 January 2020	12 September 2019
Identification of polycomb repressive complex 1 and 2 core components in hexaploid bread wheat.	10 February 2020	13 September 2019
Molecular cytological analysis of alien introgressions in common wheat lines derived from the cross of *TRITICUM AESTIVUM* with *T. kiharae*.	26 March 2020	6 September 2019
Dynamical climatic model for time to flowering in *Vigna radiata*	30 March 2020	4 September 2019
Key metabolites associated with the onset of flowering of guar genotypes (*Cyamopsis tetragonoloba* (L.) Taub).	19 May 2020	9 September 2019
Genetic diversity of *SAD* and *FAD* genes responsible for the fatty acid composition in flax cultivars and lines.	28 May 2020	27 September 2019
Effects of *Rht17* in combination with *Vrn-B1* and *Ppd-D1* alleles on agronomic traits in wheat in black earth and non-black earth regions.	15 June 2020	4 September 2019
Genetic variability of spelt factor gene in *Triticum* and *Aegilops* species.	28 May 2020	10 September 2019
The mechanism of potato resistance to *Globodera rostochiensis*: comparison of root transcriptomes of resistant and susceptible *Solanum phureja* genotypes.	31 January 2020	3 October 2019
Genome-wide association study in accessions of the mini-core collection of mungbean (*Vigna radiata*) from the World Vegetable Gene Bank (Taiwan).	30 April 2020	13 September 2019

## Footnotes

1 The received date is when an article is submitted for peer review.
